# The mediating role of attachment and mentalising in the relationship between childhood maltreatment, self-harm and suicidality

**DOI:** 10.1016/j.chiabu.2022.105576

**Published:** 2022-06

**Authors:** Maria Stagaki, Tobias Nolte, Janet Feigenbaum, Brooks King-Casas, Terry Lohrenz, Peter Fonagy, Personality and Mood Disorder Research Consortium, P. Read Montague

**Affiliations:** a Division of Psychiatry, University College London, London, United Kingdom; b Wellcome Trust Centre for Neuroimaging, University College London, London, United Kingdom; c Anna Freud National Centre for Children and Families, London, United Kingdom; d Research Department of Clinical, Educational and Health Psychology, University College London, London, United Kingdom; e Fralin Biomedical Research Institute, Department of Psychology, Virginia Tech, Roanoke, VA, United States of America; f Department of Physics, Virginia Tech, Blacksburg, VA, United States of America; g Department of Psychiatry and Behavioral Medicine, Virginia Tech Carilion School of Medicine, Virginia Tech, Roanoke, VA, United States of America

**Keywords:** Non-suicidal self-injury (NSSI), Suicidality, Childhood trauma, Attachment, Mentalising, Structural equation model

## Abstract

**Background:**

Although the relationship between childhood maltreatment, self-harm and suicidality is well-established, less is known about the mediating mechanisms explaining it. Based on a developmental mentalisation-based theoretical framework, childhood adversity compromises mentalising ability and attachment security, which in turn increase vulnerability to later stressors in adulthood.

**Objective:**

This study aimed to investigate the role of attachment and mentalising as potential mechanisms in the relationship between childhood maltreatment, self-harm and suicidality.

**Participants and setting:**

We recruited 907 adults from clinical and community settings in Greater London.

**Methods:**

The study design was cross-sectional. Participants completed self-report questionnaires on retrospectively rated childhood trauma, and current attachment to the romantic partner, mentalising, self-harm, suicidal ideation and attempt. We used structural equation modelling to examine the data and conceptualized childhood maltreatment as a general factor in a confirmatory bifactor model.

**Results:**

The results showed that childhood maltreatment was both directly associated with self-harm and suicidality and indirectly via the pathways of attachment and mentalising.

**Conclusions:**

These findings indicate that insecure attachment and impaired mentalising partially explain the association between childhood maltreatment, self-harm and suicidality. Clinically, they provide support for the potential of mentalisation-based therapy or other psychosocial interventions that aim to mitigate the risk of self-harm and suicidality among individuals who have experienced childhood maltreatment via increasing understanding of self and other mental states.

## Highlights

• Although the relationship between childhood maltreatment, self-harm and suicidality is well-established, less is known about the mediating mechanisms explaining it.
• We employed structural equation modelling in a large clinical and community sample of 907 adults, consisting largely of individuals with personality disorders and used a novel approach to conceptualise childhood maltreatment as a general factor in a confirmatory bifactor model while studying this relationship.
• We found that insecure attachment and compromised mentalising partially mediate this association and discuss these results within a mentalisation-based framework.

## 1 Introduction

### 1.1 Suicidality, self-harm and childhood maltreatment

Suicide is a major public health problem and its prevention is prioritised by the World Health Organization, 2014, World Health Organization, 2019. Suicide attempt, ideation and self-harm are strong predictors of suicidal death (Asarnow et al., 2011; Klonsky et al., 2016; Wilkinson et al., 2011). Suicide attempt and suicidal ideation were defined in this notion as suicidality, given that they frequently co-occur (Rogers et al., 2018). Self-harm, also termed non-suicidal self-injury (NSSI), was considered as a distinct concept, as it lacks conscious suicidal intent (Nock & Favazza, 2009).

Childhood maltreatment, understood as abuse or neglect which afflicts the child's health or dignity (Butchart et al., 2006), is a well-established risk factor for suicidality and self-harm (Angst et al., 2014; Chen et al., 2019; Lee, 2015; Yates et al., 2008). Meta-analyses have shown that survivors of childhood maltreatment are two to three times more likely to engage in those behaviors (Angelakis et al., 2019; Liu et al., 2017; Liu et al., 2018; Zatti et al., 2017).

Although the relationship between childhood maltreatment, self-harm and suicidality is well-documented, less is known about the mediating mechanisms underlying it. Investigating those mechanisms is critical, as they can form targets for effective preventative or therapeutic interventions that could mitigate the increased risk of suicide among childhood trauma survivors. We aimed to shed light on two potential mechanisms by exploring the mediating role of mentalising and romantic attachment in the relationship between childhood maltreatment, self-harm and suicidality in a large clinical and community sample.

### 1.2 Theoretical framework

This study was based on a developmental attachment- and mentalisation-based model (Bateman & Fonagy, 2012). Attachment theory posits that the child's early experiences with the caregiver influence the child's relationships across their lifespan, including those with romantic partners (Ainsworth et al., 1978; Bowlby, 1969; Bretherton & Munholland, 2008). Specifically, through early experiences children develop certain beliefs and expectations (working models) about themselves and others which shape their behavior in adult romantic relationships (Fraley & Shaver, 2000; Hazan & Shaver, 1987). Those working models of attachment remain relatively stable throughout life, but can be influenced by major life events (Bowlby, 1969; Fraley & Shaver, 2000). This has been supported by some longitudinal studies (McConnell & Moss, 2011; Waters et al., 2000; Zayas et al., 2011).

Mentalising is the ability to understand the self and others in terms of thoughts, behaviors and emotions (Fonagy et al., 2002). It is optimally developed in the context of secure attachment relationships with a caregiver and a benign wider social network that respect and attend to the child's mental states (Fonagy & Target, 1997; Luyten et al., 2020).

Mentalising influences psychic development and entry to the social world, as it confers epistemic trust, the attitude needed to benefit from learning opportunities from others (Bateman & Fonagy, 2016; Fonagy et al., 2015; Fonagy et al., 2017). According to Fonagy and colleagues, mentalising serves an evolutionary role in identifying trustworthy information sources (Fonagy & Allison, 2014). Extending the theory of natural pedagogy (Csibra & Gergely, 2011), they suggested that social learning occurs when the individual feels recognised and understood, which generates attention, suspends natural vigilance and signals that the subsequent information is relevant and should be accommodated in existing knowledge schemata (Fonagy et al., 2019; Fonagy & Allison, 2014; Sperber et al., 2010).

Effective mentalising is reduced in individuals that have experienced childhood adversity, such as patients with Borderline Personality Disorder (BPD), possibly due to the impact of trauma on cognitive functioning (Berthelot et al., 2015; Ensink et al., 2017; Germine et al., 2015; Lassri et al., 2018; Simon et al., 2019; Tényi & Czéh, 2019). Mentalising impairments may be an adaptation to an untrustworthy social environment, where regarding others' mental states as suspect or malintended serves short-term survival (McCrory & Viding, 2015). However, this strategy is detrimental in the long-term, as it precludes the individual from effective social learning and engenders an epistemic disadvantage, with profound consequences for the development of affect regulatory capacities (Hanson et al., 2017).

Moreover, unresponsive caregiving in intra-familiar maltreating contexts often influences the development of insecure attachment, which jeopardizes the child's sense of safety in exploring and interpreting mental states in the context of intimate relationships (Bowlby, 1969; Ensink et al., 2017; Granqvist et al., 2017; Mikulincer & Shaver, 2016). Both insecure attachment and impaired mentalising are expected to undermine interpersonal functioning and affect regulation (Bateman & Fonagy, 2012; Fonagy & Luyten, 2016). In the absence of mentalised sociocognitive mechanisms and interpersonal resources, overwhelming feelings caused by relationship stress can lead to body-centered emotion regulation through self-destructive behavior (Badoud et al., 2015; Bateman & Fonagy, 2004; Bleiberg et al., 2012; Borelli et al., 2018). Additionally, failure to attend others' mental states can also generate a sense of isolation, prompting self-harm and suicidality as an attempt to reconnect and manipulate others' behaviors (Allen, 2011; Luyten et al., 2020; Rossouw & Fonagy, 2012).

Furthermore, it is proposed that negative mental state attributions to the maltreating figure are often internalised and incorporated into the victim's self-image (“You must feel that I am a bad person to treat me like this”, therefore “I am a bad person”), despite them not originating in genuine self-experience (Fonagy & Target, 1997). These ambiguous mental states, designated as “alien-self” experiences, are associated with low self-esteem and intense shame, and endanger the individual's sense of integrity (Forrester et al., 2017; Lopez-Castro et al., 2019). In a desperate attempt to maintain mental stability and externalise unmentalised self-experiences, individuals may endeavor to control others' minds and display self-injurious or other escape behavior (Fonagy & Bateman, 2016).

### 1.3 Empirical evidence

The relationship between insecure attachment, ineffective mentalising and childhood maltreatment has been well-documented (Chiesa & Fonagy, 2014; Duval et al., 2018; Espeleta et al., 2017; Grady et al., 2019; Raby et al., 2017; Weijers et al., 2018; Zietlow et al., 2017), as well as the link between insecure attachment and self-injurious and suicidal behaviors (Wrath & Adams, 2019; Zortea et al., 2019). However, only a few studies have investigated the link between attachment to the romantic partner or mentalising and adult self-harm or suicidality, yielding mixed results which require further research (Duñó et al., 2009; Fonagy et al., 2016; Grunebaum et al., 2010; Hatkevich et al., 2019; Levesque et al., 2010; Levi-Belz et al., 2013; Smith et al., 2012).

Longitudinal evidence has also shown that anxious attachment to the caregiver mediated the relationship between child maltreatment and adult self-harm (Martin et al., 2017). Nevertheless, there is a research gap, insofar as no studies have explored romantic attachment or mentalising as unique or combined mediators in the relationship under investigation, despite the theoretical and empirical evidence that both factors contribute to the development of self-injurious and suicidal behavior in adulthood.

### 1.4 Aims and hypotheses

We aimed to bridge the research gap by investigating the mediating role of current romantic attachment and mentalising in the pathway from childhood maltreatment to self-harm and suicidality with structural equation modelling (SEM). We used a large cross-sectional sample of individuals with borderline and antisocial personality disorder, depression and anxiety and healthy control participants.

**Fig. 1:**
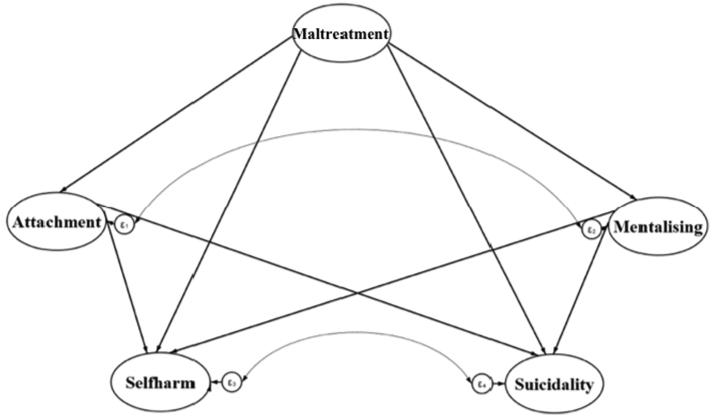
Proposed theoretical model of relationships between childhood maltreatment, insecure attachment, impaired mentalising, self-harm and suicidality. All relationships are hypothesized to be positive. Straight arrows represent paths and curved arrows indicate covariance, modelled as correlated error terms (*ε*).

We predicted that attachment and mentalising would mediate the pathway between childhood maltreatment and self-harm as well as between childhood maltreatment and suicidality. We also hypothesized that childhood maltreatment would have a direct positive association to self-harm and suicidality. Fig. 1 depicts the proposed theoretical model.

Attachment and mentalising were theorized as interrelated but not causally related, based on a) the theoretical proposition that attachment and mentalising cannot be placed on a causal chain, but rather reflect a similar underlying construct from different starting points (Fonagy & Bateman, 2016), and b) the empirical findings of Huang et al. (2020) which indicated their bidirectional relationship as mediators in the link between childhood maltreatment and PTSD. Although the latter explored only the combined effects of attachment and mentalising, we aimed to expand this approach by considering attachment and mentalising as separate but interrelated pathways, so as to investigate not only their combined effect but also the proportion of the effect explained by each construct.

We also explored separately the pathways from childhood maltreatment to self-harm and suicidality, based on findings indicating that childhood maltreatment impacts those behaviors through differential pathways (Kaess, 2019; Paul & Ortin, 2019). Nevertheless, self-harm and suicidality were theorized as interrelated phenomena, given their robust association (Nock et al., 2006; Whitlock et al., 2006).

## 2 Methods

### 2.1 Participants

We recruited 907 participants from 3 different sources in Greater London: a) 399 patients with BPD and 66 with antisocial personality disorder (ASPD) referred from personality disorder clinical services, b) 184 patients with depressive and anxiety disorders from NHS IAPT (Improving Access to Psychological Therapies) services and c) 258 healthy controls (HC) from community settings, such as universities or online platforms. Recruitment started at 2013 and it is still ongoing. The data has been previously used in studies with distinct research questions (Euler et al., 2019; Huang et al., 2020; Wendt et al., 2019).

Inclusion criteria were: a) aged 16–65 years old, b) fluent in English, while exclusion criteria were: a) schizophrenia diagnosis or recent psychotic episode, b) learning disability and c) neurological disorder. Inclusion criteria specifically for BPD/ASPD participants were: a) suspected/confirmed diagnosis and b) being on the waiting list for therapy or participating in a psychoeducational group. Regarding participants recruited through IAPT services, specific inclusion criteria were: a) experiencing significant depressive symptoms and b) deemed eligible for face-to-face low or high intensity IAPT therapy. Suspected/confirmed BPD/ASPD diagnosis was assessed by the research team through the Structured Clinical Interview for DSM-IV Axis II personality disorders (SCID-II; First, 2014) and the Personality Assessment Inventory-Borderline Scale (PAI-BOR; Morey, 1991).

### 2.2 Procedure

The study design was cross-sectional. BPD/ASPD/HC attended 2 testing appointments at University College London, where they completed computerised behavioral social interaction tasks, self-report questionnaires and personality interviews. Participants with depressive and anxiety disorders participated in a remote, online version of this study, completing the same self-report questionnaires, one of the behavioral social interaction tasks and no personality interviews. All participants provided signed informed consent. Participants were compensated with £10 per hour for their participation with the possibility of additional performance-related compensation for the behavioral task component(s).

The dataset was derived from the larger study “Probing Social Exchanges- A Computational Neuroscience Approach to the Understanding of Borderline and Anti-Social Personality Disorder” and the sub-study “Major Depressive Disorder- A Computational Psychiatry Approach: Understanding the Social Brain in Healthy Volunteers and People with Psychological Difficulties”. Ethical approval for the BPD/ASPD/ HC study was acquired from the Research Ethics Committee (REC) of Wales (REC number: 12/WA/0283) and for the reduced sub-study from the London Queen Square REC (REC number: 16/LO/0077).

### 2.3 Measurement instruments

#### 2.3.1 Childhood maltreatment

Childhood maltreatment was measured with the Childhood trauma questionnaire (CTQ) (Bernstein et al., 1994), a 28-item self-report retrospective inventory. Respondents retrospectively indicated on a five-point Likert scale the frequency of certain traumatic events in childhood or adolescence (1 = never, 5 = very often). The CTQ includes five subscales, measuring physical, sexual and emotional abuse as well as physical and emotional neglect (Bernstein & Fink, 1998). Each subscale contains 5 items and scores range from 5 (absence of maltreatment) to 25 (severe maltreatment history). The questionnaire has high internal consistency, test-retest reliability and established validity (Kongerslev et al., 2019; Thombs et al., 2009). In the current sample, Cronbach's alpha was 0.90 for emotional and physical abuse, 0.96 for sexual abuse, 0.76 for physical neglect and 0.91 for emotional neglect, showing high internal consistency (Tavakol & Dennick, 2011).

#### 2.3.2 Romantic attachment

Romantic attachment was measured with the Experiences in Close Relationships Scale (ECR-R; Fraley et al., 2000). Participants who were not currently involved in a romantic relationship were asked to respond based on how they felt in their more recent relationship. Individuals with no experience of romantic relationships were asked to imagine what they would be like in a relationship. Participants indicated in a seven-point Likert scale the extent of agreement or disagreement with 36 statements (1 = strongly agree, 7 = strongly disagree).

Attachment is measured by two higher-order dimensions: anxiety over abandonment and avoidance of intimacy. Elevated subscale scores denote higher attachment anxiety or avoidance. The psychometric properties of the ECR-R two-factor structure are well-established (Alonso-Arbioll et al., 2007; Sibley et al., 2005). In the current study, Cronbach's alpha for anxiety subscable was 0.93 and for avoidance subscale 0.94, demonstrating excellent internal consistency (Tavakol & Dennick, 2011).

#### 2.3.3 Mentalising

Mentalising was assessed with the Reflective Functioning Questionnaire (RFQ; Fonagy et al., 2016), a 54-item self-report inventory capturing the ability to understand mental states of the self and others. Participants endorse a series of statements on a seven-point Likert scale (1 = strongly disagree, 7 = strongly agree). The questionnaire yields two dimensional subscales: Uncertainty subscale (hypomentalising) reflecting an extreme lack of knowledge about mental states and Certainty subscale (hypermentalising) indicating excessive certainty about mental states. Higher scores on the certainty subscale and lower scores on the uncertainty subscale indicate better mentalising capacity (Spitzer et al., 2021). The questionnaire has excellent test-retest reliability and internal consistency, also demonstrated in the current sample both for Certainty (*α* = 0.90) and Uncertainty subscale (*α* = 0.91) (Badoud et al., 2015; Fonagy et al., 2016).

#### 2.3.4 Self-harm

Self-harm was measured as a latent construct based on three items using Confirmatory Factory Analysis (CFA): 1) “What is the greatest number of times, in any one year, that you have tried to hurt yourself as described in question 9?” assessed on a six-point Likert scale (1 = never happened, 6 = happened so many times that I can't give a number), from the Drugs, Alcohol and Self-Injury Questionnaire (DASI; Cassels et al., 2018; Wilkinson et al., 2018). This item refers to the previous question “Have you ever tried to hurt yourself on purpose, without trying to kill yourself?”, which was not included in the CFA, due to not providing additional information. 2) “When I am upset, I typically do something to hurt myself”, assessed on a four-point Likert scale (1 = false, 4 = very true), from the Personality Assessment Inventory-Borderline Scale (PAI-BOR; Morey, 1991). 3) “Deliberately tried to physically hurt yourself in anger or desperation” measured on a four-point Likert scale (1 = false, 4 = very true), from the Life History of Aggression questionnaire (LHA; Coccaro et al., 1997).

#### 2.3.5 Suicidality

Suicidality, defined as suicidal thoughts and attempts, was measured as a latent construct based on three items using CFA: 1) “Indicate in the last seven days, how much you were distressed by thoughts of ending your life” and 2) “Indicate in the last 7 days, how much you were distressed by thoughts of death or dying”, assessed on a five-point Likert scale (1 = not at all, 5 = extremely) from the Brief Symptom Inventory (BSI; Derogatis, 1992). 3) “Deliberately tried to end your life or kill yourself in anger or desperation” measured on a four-point likert scale (1 = false, 4 = very true), from the LHA; Coccaro et al., 1997). High correlation between the first two items ensured that the second referred to suicidal thoughts and not a different construct, such as death or health anxiety (*rs* = 0.79, *p* < 0.001).

### 2.4 Data analysis

We used the recommended two-step approach of structural equation modelling (SEM) (Anderson & Gerbing, 1988). First, the measurement model was tested with CFA. Adequate factor loadings and goodness of fit provided a good foundation for proceeding to the second step of testing the full structural equation model of hypothesized paths and covariances (Kline, 2016).

We investigated bivariate correlations between variables using Spearman rank coefficient and explored potential gender differences in the study variables with Mann-Whitney *U* tests, given the non-normality of variables. We aimed to control for age, gender and education level, due to their expected relationship with the variables of interest, to eliminate alternative causal pathways between variables (Barbosa et al., 2014; Lang & Sharma-Patel, 2011; Mickelson et al., 1997; Quek et al., 2017).

#### 2.4.1 Model specification

The following latent constructs and their interrelations were included in the measurement model: childhood maltreatment, attachment, mentalising, self-harm and suicidality. Attachment was measured as a latent factor derived from avoidance and anxiety subscales and mentalising through uncertainty and certainty subscales. Self-harm and suicidality were derived from the three variables described above which were standardised, to account for the different scaling between them.

Regarding the conceptualization of childhood maltreatment, we observed high intercorrelations between CTQ subscales, suggesting that a bifactor model might constitute a more parsimonious explanation (Spinhoven et al., 2014). Bifactor modelling is well-suited for representing multidimensional constructs and has been used to study the dimensional structure of the CTQ, showing good fit to the data (Chen et al., 2012; Spinhoven et al., 2014). Due to the debate in the literature regarding the interpretability of specific factors, we focused on general “childhood maltreatment” (Reise et al., 2010). Seven CTQ items indicating absence of maltreatment (e.g. “I felt loved”) were reverse coded (1 = 5, 2 = 4, 3 = 3, 5 = 1, 4 = 2).

In the structural equation model, covariances between latent constructs were replaced by paths. However, as the relationships between attachment and mentalising and between self-harm and suicidality were assumed not to be causal, they were modelled as correlated residuals.

#### 2.4.2 Model estimation

We estimated the model with maximum likelihood method (ML; Kline, 2016), which employes listwise deletion of missing values, and investigated multivariate normality assumption based on both graphs and multivariate normality tests. All graphs and most tests (Mardia's multivariate skewness test: *χ2*(df = 20) = 311.980, Henze-Zirkler consistent test *χ2*(df = 1) = 313.090 and Doornik-Hansen omnibus test *χ2*(df = 8) = 449.299, *p* < 0.001) suggested violation of multivariate normality assumption (except for Mardia's multivariate kurtosis test: *χ2*(df = 1) = 0.103, *p* = 0.748). Therefore, Sattora-Bentler adjustment was employed, which provides standard errors robust to nonnormality and produces improved goodness of fit indices (Chou et al., 1991; Curran et al., 1996; Satorra & Bentler, 1994).

To judge an acceptable goodness of fit, the following indices were considered more appropriate: Root Mean Square Error of Approximation (RMSEA), Comparative Fit Index (CFI) and Tucker-Lewis Index (TLI). Values smaller than 0.060 for RMSEA and values higher than 0.95 for CFI and TLI indicate excellent model fit (Byrne, 2010; Hu & Bentler, 1999; Kline, 2016), while CFI and TLI values ≥0.90 show acceptable model fit (Brown, 2006). The chi-squared statistic was also reported, but was judged to be less informative, due to its sensitivity to sample size (Byrne, 2010). We examined direct and indirect effects to investigate the mediation hypothesis. Data analysis was conducted using the Statistical Package for the Social Sciences (SPSS) and STATA version 15.

## 3 Results

### 3.1 Descriptive statistics and exploratory analyses

The sample included 907 participants, with mean age 30.7 years (*SD* = 10.3).The majority was female (*n* = 628, 69.2%) and White in ethnicity (*n* = 604, 66.6%). Almost half were diagnosed with BPD (*n* = 399, 44.0%) and almost a third were HC participants (*n* = 258, 28.4%). Moreover, 20.3% had depressive and anxiety disorders (*n* = 184) and only 7.3% (*n* = 66) ASPD (personality disorder diagnosis was established using SCID-II). The sample was heterogeneous in terms of employment status and education levels (Table 1).

**Table 1:** Demographic and clinical characteristics of the sample.

Variable		Total sample (*N* = 907)
Age (years) Range 18–65		30.7 (10.3)
Gender	Male	272 (30.0%)
	Female	628 (69.2%)
Ethnicity	White	604 (66.6%)
	Black	101 (11.1%)
	Asian	83 (9.2%)
	Mixed/ other	108 (11.9%)
Employment status	Employed	362 (39.9%)
	Unemployed	356 (39.3%)
	Student	148 (16.3%)
	Other	28 (3.1%)
Education level	NVQ 1/ GCSE <5 A*-C	74 (8.2%)
	NVQ 2/ GCSE ≥5 A*-C	166 (18.3%)
	A-level/ NVQ 3	252 (27.8%)
	Higher education	232 (25.6%)
Education level	Postgraduate education	82 (9.0%)
	No qualifications	57 (6.3%)
	Other qualification	33 (3.6%)
Subsample	Borderline personality disorder	399 (44.0%)
	Antisocial personality disorder	66 (7.3%)
	Healthy controls	258 (28.4%)
	Depressive or anxiety disorder (IAPT-referred)	184 (20.3%)

*Note.* Data are N (%) or mean (SD). NVQ = National Vocational Qualification. GCSE = General Certificate of Secondary Education. GCSE <5 A*-C: achievement of less than five A*-C grades at GSCE. GCSE ≥5 A*-C: achievement of five or more A*-C grades at GSCE. Missing data: age *n* = 7 (0.8%), gender n = 7 (0.8%), ethnicity *n* = 11 (1.2%), educational level n = 11 (1.2%), employment status *n* = 13 (1.4%). Demographic data were collected by self-report.

Table 2 shows descriptive statistics and bivariate correlations between study and potential control variables. Strong evidence demonstrated that all study variables were correlated (*p* < 0.001). There was also evidence indicating an association between age, education and various study variables, as well as gender differences in mentalising uncertainty, attachment, self-harm and suicide attempt. Thus, we included age, education level and gender as covariates in the model (Table 2, Table 3).

**Table 2:** Means, standard deviations and correlations between study variables.

Variables	1	2	3	4	5	6	7	8	9	10	11	12	13	14	15	16	17
1. Mentalising Uncertainty	1																
2. Mentalising Certainty	−0.67⁎⁎	1															
3. Attachment Anxiety	0.51⁎⁎	−0.43⁎⁎	1														
4. Attachment Avoidance	0.38⁎⁎	−0.34⁎⁎	0.42⁎⁎	1													
5. Self-harm1	0.49⁎⁎	−0.35⁎⁎	0.54⁎⁎	0.25⁎⁎	1												
6. Self-harm2	0.55⁎⁎	−0.41⁎⁎	0.62⁎⁎	0.35⁎⁎	0.75⁎⁎	1											
7. Self-harm3	0.54⁎⁎	−0.40⁎⁎	0.58⁎⁎	0.29⁎⁎	0.86⁎⁎	0.78⁎⁎	1										
8. Suicidality1 (ideation)	0.43⁎⁎	−0.25⁎⁎	0.45⁎⁎	0.29⁎⁎	0.52⁎⁎	0.57⁎⁎	0.55⁎⁎	1									
9. Suicidality2 (ideation)	0.46⁎⁎	−0.29⁎⁎	0.49⁎⁎	0.31⁎⁎	0.52⁎⁎	0.56⁎⁎	0.56⁎⁎	0.79⁎⁎	1								
10. Suicidality3 (attempt) range 0–5	0.51⁎⁎	−0.37⁎⁎	0.49⁎⁎	0.33⁎⁎	0.66⁎⁎	0.67⁎⁎	0.73⁎⁎	0.59⁎⁎	0.53⁎⁎	1							
11. Emotional Abuse A)	0.34⁎⁎	−0.20⁎⁎	0.45⁎⁎	0.30⁎⁎	0.50⁎⁎	0.46⁎⁎	0.53⁎⁎	0.44⁎⁎	0.45⁎⁎	0.52⁎⁎	1						
12. Physical Abuse	0.19⁎⁎	−0.10⁎⁎	0.24⁎⁎	0.22⁎⁎	0.26⁎⁎	0.26⁎⁎	0.28⁎⁎	0.30⁎⁎	0.31⁎⁎	0.37⁎⁎	0.66⁎⁎	1					
13. Sexual Abuse	0.19⁎⁎	−0.08⁎⁎	0.28⁎⁎	0.20⁎⁎	0.29⁎⁎	0.26⁎⁎	0.30⁎⁎	0.24⁎⁎	0.26⁎⁎	0.35⁎⁎	0.48⁎⁎	0.45⁎⁎	1				
Variables	1	2	3	4	5	6	7	8	9	10	11	12	13	14	15	16	17
14. Emotional Neglect	0.29⁎⁎	−0.23⁎⁎	0.34⁎⁎	0.35⁎⁎	0.35⁎⁎	0.36⁎⁎	0.36⁎⁎	0.33⁎⁎	0.31⁎⁎	0.39⁎⁎	0.71⁎⁎	0.52⁎⁎	0.31⁎⁎	1			
15. Physical Neglect	0.24⁎⁎	−0.18⁎⁎	0.33⁎⁎	0.30⁎⁎	0.31⁎⁎	0.33⁎⁎	0.33⁎⁎	0.30⁎⁎	0.34⁎⁎	0.39⁎⁎	0.64⁎⁎	0.56⁎⁎	0.37⁎⁎	0.72⁎⁎	1		
16. Age	0.01	0.01	−0.03	0.02	−0.10⁎	−0.05	−0.07	0.01	0.02	0.04	0.07	0.12⁎	0.11⁎	0.12⁎	0.12⁎	1	
17. Education level	−0.10⁎	0.08†	−0.05	−0.08†	0.02	−0.02	−0.01	−0.03	−0.01	−0.11⁎	−0.03	−0.11⁎	−0.04	−0.10⁎	−0.18⁎	0.01	1
Mean	21.27	18.85	4.12	3.51	1.58	1.11	2.52	1.01	1.43	1.56	13.43	9.05	8.57	14.20	9.22	30.7	4.73
SD	15.00	13.92	1.57	1.41	1.37	1.20	2.14	1.38	1.56	1.80	6.58	5.60	6.17	6.09	4.37	10.3	1.56
Range	0–73	0–73	1–7	1–7	0–4	0–3	0–5	0–4	0–4	0–5	5–25	5–25	5–25	5–25	5–25	16–65	1–7

*Note.* Missing data: Uncertainty, Certainty *n* = 113 (12.5%), Anxiety *n* = 85 (9.4%), Avoidance *n* = 79 (8.7%), selfharm1 *n* = 42 (4.6%), selfharm2 *n* = 52 (5.7%), selfharm3 *n* = 51 (5.6%), suicidality1 *n* = 78 (8.6%), suicidality2 *n* = 80 (8.8%), suicidality3 *n* = 53 (5.8%), emotional abuse, emotional neglect, physical neglect *n* = 96 (10.6%), physical abuse *n* = 95 (10.5%), sexual abuse *n* = 101 (11.1%), age n = 7 (0.8%), educational level n = 11 (1.2%).

⁎⁎ *p* < 0.001.

⁎ *p* < 0.01.

† *p* < 0.05.

**Table 3:** Gender differences in study variables

	Female (N = 628)		Male (*N* = 272)			
Variables	M	SD	M	SD	z	*p*-Value
Mentalising uncertainty	21.85	15.03	19.57	14.48	−2.03	**0.04**
Mentalising certainty	18.47	13.54	19.88	14.66	−1.11	0.27
Attachment anxiety	4.33	1.55	3.61	1.53	−5.99	**<0.001**
Attachment avoidance	3.61	1.47	3.25	1.23	−2.97	**0.003**
Self-harm1	1.77	1.35	1.14	1.32	−6.11	**<0.001**
Self-harm2	1.27	1.23	0.74	1.07	−6.04	**<0.001**
Self-harm3	2.81	2.12	1.85	2.01	−5.99	**<0.001**
Suicidality1 (ideation)	1.03	1.38	0.96	1.36	−0.97	0.33
Suicidality2 (ideation)	1.47	1.57	1.30	1.50	−1.53	0.13
Suicidality3 (attempt)	1.69	1.82	1.23	1.70	−3.75	**<0.001**
Emotional abuse	14.31	6.63	11.39	5.99	−1.12	0.27
Physical abuse	9.03	5.56	9.07	5.71	−0.15	0.88
Sexual abuse	9.06	6.45	7.41	5.27	−1.53	0.13
Emotional neglect	14.60	6.07	13.27	6.06	−0.09	0.93
Physical neglect	9.31	4.41	9.04	4.32	−0.30	0.77

### 3.2 Confirmatory bifactor model of childhood maltreatment

**Fig. 2:**
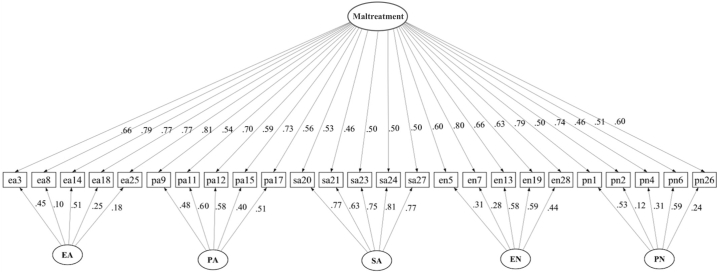
Confirmatory Bifactor Model of Childhood maltreatment measured by the Childhood Trauma Questionnaire (*N* = 716). Items load on a general factor (Childhood Maltreatment) and specific group factors (EA = Emotional Abuse, PA = Physical Abuse, SA = Sexual Abuse, EN = Emotional Neglect, PN=Physical Neglect). All factor loadings are standardised and statistically significant (p < 0.001). Rectangles represent observed variables and ovals reflect latent constructs. Covariances between observed variables are not displayed.

We employed a novel approach in the conceptualization of childhood maltreatment with a confirmatory bifactor model, which has only been adopted by few previous studies (Hollerbach et al., 2018; Spinhoven et al., 2014). In a confirmatory bifactor model each item loads on two factors; one general and one specific factor (Reise et al., 2010). The two loading estimates depicted in Fig. 2 reflect the relation of each item to the general ‘maltreatment’ factor and the allocated specific factor (Dunn & McCray, 2020). The bifactor model allowed us to investigate childhood maltreatment as an overarching construct while recognising its multidimensional nature and accounting for the shared variance among different but largely overlapping types of abuse and neglect.

Table 4 presents CTQ items included in the bifactor model which is shown in Fig. 2. Strong evidence showed that all items loaded on the general and corresponding specific factors (p < 0.001). Most item loadings on the general “maltreatment” factor were stronger (0.46–0.81) than loadings on specific physical, emotional abuse and neglect factors (0.10–0.59) with exception to sexual abuse subscale. We included the general “maltreatment” factor in the measurement model.

**Table 4:** Means and standard deviations of CTQ items in the Bifactor Model.

Variables	M	SD
Emotional abuse (EA):		
3. Called names by family	2.76	1.50
8. Parents wished was never born	2.37	1.50
14. Family said hurtful things	2.99	1.45
18. Felt hated by family	2.65	1.62
25. Was emotionally abused	2.66	1.66
Physical abuse (PA):		
9. Hit hard enough to see doctor	1.44	1.03
Variables	M	SD
11. Hit hard enough to leave bruises	2.05	1.44
12. Punished with hard objects	2.08	1.47
15. Was physically abused	2.01	1.51
17. Hit badly enough to be noticed	1.48	1.13
Sexual abuse (SA):		
20. Was touched sexually	1.85	1.41
21. Hurt if didn't do something sexual	1.45	1.12
23. Made to do sexual things	1.73	1.32
24. Was molested	1.73	1.36
27. Was sexually abused	1.81	1.46
Variables	M	SD
Emotional neglect (EN):		
5. Made to feel important (rev)	2.68	1.48
7. Felt loved (rev)	2.56	1.36
13. Was looked out for (rev)	2.80	1.40
19. Family felt close (rev)	3.10	1.40
28. Family was source of strength (rev)	3.07	1.46
Physical neglect (PN):		
1. Not enough to eat	1.79	1.17
2. Got taken care of (rev)	3.61	1.41
4. Parents were drunk or high	1.63	1.20
6. Wore dirty clothes	1.46	1.01
26. Got taken to a doctor (rev)	1.97	1.29

*Note.* The table was adapted by Bernstein et al. (1998). Range of all items: 1–5 (1 = never true, 2 = rarely true, 3 = sometimes true, 4 = often true, 5 = very often true) rev = reverse scored items. Missing data: Ea3, en7, ea8, pa9, pa11 and pa15: *n* = 91, 10.1%. Pn1, pn2, pn6, pa12 and en28: *n* = 92, 10.0%. Pn4, pa17, en19, sa20, sa21 and sa27: *n* = 94, 10.4%. En5, en13, ea14, ea18, ea25 and pn26 *n* = 93, 10.3%. Sa23 and sa24: n = 94, 10.4%.

### 3.3 Confirmatory factor analysis and structural equation model

The measurement model, including all possible correlations between latent factors, showed marginal fit (RMSEA = 0.053, CFI = 0.927, TLI = 0.917, *χ2*(df = 615) = 1835.823, *p* < 0.001). To improve model fit, we examined modification indices and allowed the following covariances between observed variables with shared components and meaningful theoretical relations (Byrne, 2005): items 21, 23 from sexual abuse subscale denoting sexual coercion, items 9, 17 from physical abuse subscale indicating external intervention due to bodily injuries and items 5, 7 from emotional neglect subscale reflecting fulfillment of affectional needs (see Table 4 for description of CTQ items). Two items forming the suicidality construct and reflecting suicidal ideation were freed to covary, due to the specific time frame “last 7 days”, which is an additional cause of covariance not accounted for by the latent variable suicidality.

**Fig. 3:**
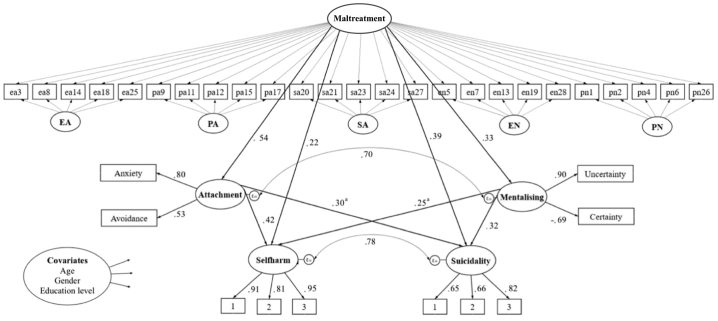
Structural equation model of relations between childhood maltreatment, mentalising, attachment, self-harm and suicidality (*N* = 716). Covariates were allowed to predict all endogenous variables in the model. Coefficients are standardised and statistically significant (*p* < 0.001), ^a^*p* = 0.001. Confidence Intervals are reported in Table 5. EA = Emotional Abuse, PA = Physical Abuse, SA = Sexual Abuse, EN = Emotional Neglect, PN=Physical Neglect. Rectangles represent observed variables, ovals reflect latent constructs and small circles indicate error terms (residuals). Straight arrows represent paths and curved two-headed arrows indicate covariance. Covariances between observed variables are not displayed.

The revised CFA model demonstrated acceptable fit, providing adequate foundation to test structural relationships: RMSEA = 0.044, CFI = 0.949, TLI = 0.941, whilst the sample size dependent *χ2* test indicated less adequate model fit: *χ2*(df = 611) = 1469.295, *p* < 0.001. Latent factors showed moderate to strong correlations (0.34–0.89; *p* < 0.001*, p* = 0.001). Items loaded moderately to strongly on latent factors (0.53–0.95; *p* < 0.001), as shown in Fig. 3.

The structural equation model, depicted in Fig. 3, showed identical fit indices with the measurement model. Strong evidence demonstrated positive associations between model constructs, as well as between the residuals of attachment and mentalising and of self-harm and suicidality (*p* < 0.001).

### 3.4 Mediation analysis examining indirect effects

Table 5 presents standardised direct and indirect effects from the proposed mediation model. There was strong evidence of indirect effects of childhood maltreatment on self-harm and suicidality via attachment and mentalising, both as unique and combined mediators. Attachment explained a larger proportion of the indirect effect of childhood maltreatment on self-harm and suicidality. Direct effects of maltreatment on suicidality and self-harm remained statistically significant (*p* < 0.001), indicating partial mediation. The structural model explained 68% of the variance in suicidality, 61% in self-harm, 36% in attachment and 13% in mentalising.

**Table 5:** Standardised direct and indirect effects from the proposed mediation model of the relationship between childhood maltreatment, self-harm and suicidality, controlled for age, gender and education level.

Pathway	Indirect effect	95% CI
Maltreatment–Attachment–Mentalising–Suicidality	0.27*	0.19–0.34
Maltreatment–Attachment–Suicidality	0.16 ^a^	0.06–0.27
Maltreatment–Mentalising–Suicidality	0.11*	0.05–0.16
Maltreatment–Attachment–Mentalising–Self-harm	0.31*	0.23–0.39
Maltreatment–Attachment–Self-harm	0.23*	0.12–0.34
Maltreatment–Mentalising–Self-harm	0.08 ^a^	0.03–0.14


Pathway	Direct effect	95% CI
Maltreatment–Suicidality	0.39*	0.29–0.49
Maltreatment–Self-harm	0.22*	0.13–0.31
Maltreatment–Attachment	0.54*	0.47–0.62
Maltreatment–Mentalising	0.33*	0.25–0.41
Attachment–Suicidality	0.30 ^b^	0.12–0.48
Attachment–Self-harm	0.42*	0.24–0.61
Mentalising–Suicidality	0.32*	0.17–0.46
Mentalising–Self-harm	0.25 ^b^	0.10–0.40

Note. *p < 0.001, ^a^*p* = 0.002, ^b^*p* = 0.001. CI: Confidence Interval.

## 4 Discussion

Our findings indicated that the proposed theoretical model provided a good fit to the data, supporting our hypotheses. Childhood maltreatment as an overarching construct, derived through bifactor modelling, was found to be directly associated with self-harm and suicidality and these relationships were partially mediated by insecure attachment to the romantic partner and ineffective mentalising. Further, attachment accounted for the largest proportion of the indirect effect of childhood maltreatment on both self-harm and suicidality. Overall, these findings support a model in which childhood maltreatment not only has a direct impact on self-harm and suicidality, but that social relationship difficulties as indicated by insecure attachment and mentalising in part account for the powerful, arguably causal, association between childhood maltreatment and those behaviors.

Although no other study has provided support for the combined mediating role of attachment and mentalising intervening this pathway, our results are partly consistent with previous mediation findings. For example, in a longitudinal study Martin et al. (2017) demonstrated that insecure attachment to the caregiver mediated the impact of childhood maltreatment on self-harm at age 26. Previous clinical studies have also reported the unique mediating role of attachment and mentalising in the link between childhood adversity and various forms of psychopathology (Chiesa et al., 2020; Chiesa & Fonagy, 2014; Duval et al., 2018; Hankin, 2005). We extended those findings by incorporating both factors in a single overarching framework to identify mechanisms underlying self-injurious and suicidal behavior in a mixed sample that included a large proportion of high-risk personality disordered patients.

The associations found between different pairs of variables in our model are in line with previous literature. Specifically, the direct effect of childhood maltreatment on self-harm and suicidality corresponds to previous studies establishing childhood abuse as a robust risk factor for adult self-injurious and suicidal behavior (Angelakis et al., 2019; Yates et al., 2008). The positive association between childhood trauma and adult insecure attachment was also expected based on previous longitudinal findings indicating that childhood maltreatment predicted an increased risk of insecure romantic attachment (Raby et al., 2017). Childhood maltreatment was positively associated with ineffective mentalising, as expected based on previous research establishing childhood maltreatment as a vulnerability factor for mentalising impairments (Chiesa & Fonagy, 2014; Duval et al., 2018). In our model, both insecure attachment and impaired mentalising showed a positive link to self-injurious and suicidal behavior, which is also in line with previous literature (Aaltonen et al., 2016; Badoud et al., 2015; Fonagy et al., 2016; Grunebaum et al., 2010; Hatkevich et al., 2019; Martin et al., 2017).

The results of this study shed light on the mechanisms underlying the relationship between childhood maltreatment, self-harm and suicidality, based on a developmental mentalisation-based perspective (Bateman & Fonagy, 2012). According to it, childhood maltreatment undermines secure attachment and mentalising capacity (Bateman & Fonagy, 2012; Mikulincer & Shaver, 2016). Insecure attachment and ineffective mentalising, in turn, are believed to compromise affect regulation and generate heightened epistemic vigilance, which impairs the ability to build social learning cycles and meaningfully connect to supportive social networks undermining a natural process of self-correction in relation to thoughts and feelings (Fonagy et al., 2002; Fonagy et al., 2015; Luyten et al., 2020).

Childhood trauma survivors with insecure attachment are often sensitised to feelings of abandonment or neglect (Bateman & Fonagy, 2012). Thus, stressful interpersonal experiences can activate the bio-behavioral system underpinning attachment, giving rise to overwhelming emotions which further inhibit mentalising capacity (Nolte et al., 2013). Unmentalised traumatic experiences can cause intense distress, as they are often relived as intense emotional experiences in the present. This can prompt avoidance of the associated mental experience, that is instead regulated via physical actions, including violent self-directed acts (Allen, 2011). This latter form of ineffective mentalising corresponds to what has been labelled as a ‘teleological mode’, in which actions, in this case self-injurious ones, are experienced as the sole way of managing unbearable emotions (Bateman & Fonagy, 2012). This has been supported by ecological momentary assessment studies which found that increased negative affect precedes self-injurious and suicidal behavior, especially among individuals with BPD who show elevated affective instability (Andrewes et al., 2017; Mou et al., 2018; Reichl & Kaess, 2021; Scala et al., 2018). A meta-analysis by Taylor et al. (2018) also concluded that self-harm was most commonly used as an emotion regulatory strategy serving avoidance of a negative state, while fewer people tend to endorse its interpersonal function.

The interrelation between attachment and mentalising proposed by the theoretical model was confirmed in our model. Nonetheless, it has been proposed that attachment and mentalising are inherently interdependent in childhood and thus, inclusion of two overlapping factors into a single model might be problematic (Fonagy & Bateman, 2016). This was not confirmed by our results. Both attachment and mentalising were retained in the model and explained different proportions of the indirect effect of childhood maltreatment on self-harm and suicidality, through separate pathways. A similar dual track impact of therapeutic effects on self-harm was reported in a randomized controlled trial of adolescents (Rossouw & Fonagy, 2012). This could suggest that although mentalising and attachment are interrelated in childhood, their dysfunction may represent alternative processes for initiating or maintaining self-harm and reflect somewhat separate ways social connections may manifest challenges in adulthood. This could have clinical importance suggesting that simultaneously therapeutically addressing close (attachment) relationships and ineffective mentalising may yield better outcomes in tackling self-harm than either alone.

Our results provide support for the mentalising theoretical framework, insofar as mentalising and attachment were shown to partially mediate the relationship between childhood maltreatment and self-harm and childhood maltreatment and suicidality. However, as the mediation was shown to be only partial, a portion of shared variance both between childhood maltreatment, self-harm and suicidality remained unexplained. This finding is subject to different interpretations: The measures assessing mentalising and attachment may not fully reflect the underlying constructs, alternatively or in addition other mechanisms need to be postulated to explain the effect of childhood maltreatment on suicidality and self-harm.

Such alternative mechanisms could be related to biological processes, such as the effects of childhood adversity on brain development and epigenetic modifications (Cecil et al., 2020; Labonte & Turecki, 2010), as well as psychological factors not directly accounted for in the current study, such as emotion dysregulation, self-criticism, dissociation or alexithymia (Chen et al., 2019; Euler et al., 2019; Franzke et al., 2015; Glassman et al., 2007; Titelius et al., 2018). The role of social factors, specifically decreased social support and interpersonal difficulties, has also been highlighted in previous research (Chen et al., 2019; Lemaigre & Taylor, 2019). We suggest that these broader social contextual variables may be captured by the concept of epistemic trust described above. Further research will need to explore if introducing this parameter into mediational models provides better account of how maltreatment generates self-harm and suicidality outcomes. It is also important that future studies investigate the complex interactions between biopsychosocial processes to elucidate the mechanisms fully mediating the pathway from childhood maltreatment to adult self-harm and suicidality, in light of their multifactorial nature (Sachs-Ericsson et al., 2016).

### 4.1 Limitations

Several limitations of this study warrant consideration. Our sample consisted mainly of participants of White ethnicity, female gender and personality disorders. Moreover, the participants' socioeconomic background was not assessed. This reduces the sample's representativeness and precludes us from drawing inferences for any specific clinical or community population. However, the use of a predominantly clinical sample was necessary to capture a range of severity in childhood maltreatment history, self-injurious and suicidal behaviors, as these largely occur in clinical populations of higher severity (Devi et al., 2019; Lee et al., 2007).

Secondly, our research study is cross-sectional, rendering any causal inferences between measured constructs largely speculative. Future research employing longitudinal designs would allow the investigation of temporal associations. This study also used self-report measures, which are liable to social desirability and mood state bias (Podsakoff et al., 2003). Specifically, retrospective measurement of childhood trauma, self-harm and suicidality is prone to recall bias and human memory imperfections. Nonetheless, retrospective measurement of childhood adversity has been documented as relatively accurate (Bifulco et al., 1997; Brewin et al., 1993; Hardt & Rutter, 2004).

Moreover, measuring childhood maltreatment through the CTQ did not allows us to capture different forms of childhood adversity such as parental incarceration, divorce, school violence, natural disasters or wars, which have been been linked with suicidal behaviors and can also affect attachment and psychic development in adulthood (Dube et al., 2001; Felitti et al., 1998; Green et al., 1994; Murphy et al., 2014). Therefore, it would be beneficial for future studies to investigate how attachment and mentalising potentially mediate the relationship between those different forms of adverse childhood experiences, self-harm and suicidality.

There is also evidence indicating that robust weighted least squares estimation method (WLS) is preferable to ML for Likert-scale variables with few categories (Kline, 2016; Li, 2014; Li, 2016). Thus, future studies might do well to investigate if both approaches yield comparable results.

### 4.2 Future research directions

Besides investigating alternative mediators, future research could explore the same research question while measuring the multifaceted constructs of attachment and mentalising differently. For instance, attachment can be assessed in relation to other attachment figures, such as parents, and through other measures, such as the Adult Attachment Interview (AAI), which captures disorganized attachment (George et al., 1996). Mentalising could also be measured as a relationship-specific rather than general ability through the Reflective Functioning Scale in the AAI (Fonagy et al., 1998; Luyten et al., 2012).

Moreover, replication of our findings is necessary, especially in sociodemographically heterogeneous, clinical or community samples, to facilitate generalisability of findings in the corresponding population. As there is also evidence that different childhood maltreatment types interact differently with different types of mentalising and attachment, future research should explore those complex interactions (Kristiansen et al., 2020; Raby et al., 2017).

### 4.3 Clinical implications

The current study underlines the importance of mentalising and attachment relationships as potentially effective separate treatment foci for victims of childhood maltreatment presenting with self-injurious or suicidal behavior. Evidence-based treatments that foster mentalising and attachment, such as mentalisation-based treatment, transference-focused therapy and attachment-based family therapy, can have valuable benefits for victims of childhood trauma who exhibit self-injurious and suicidal behavior or are at increased risk (Allen, 2013; Diamond et al., 2014; Diamond et al., 2016; Fonagy & Bateman, 2006; Kemberg et al., 2008). Although there is already evidence supporting the effectiveness of these treatments in reducing self-harm and suicidality for borderline personality disorder (Bateman and Fonagy, 2008, Bateman and Fonagy, 2009; Levy et al., 2007), extension of those findings measuring changes in attachment relationships and mentalising while monitoring treatment outcomes in personality disordered and other clinical and community populations in RCTs is essential. Furthermore, fostering secure attachment and mentalising capacity can be valuable for suicide prevention efforts targeting at-risk individuals with a history of childhood maltreatment. Lastly, if clinicians evaluate childhood trauma history, attachment and mentalising in clinical assessments, they could potentially better identify individuals who are at high risk of self-harm or suicidality.

## Funding

This work was supported by a 10.13039/501100000272National Institute for Health Research (NIHR) Senior Investigator Award (NF-SI-0514-10157) awarded to Peter Fonagy. The work was also supported by NIH-NIDS Grant 5R01NS092701-03, 10.13039/100000002National Institutes of Health Award (MH115221) awarded to Brooks King-Casas, the Kane Family Foundation and a 10.13039/100010269Wellcome Trust Senior Investigator Award to P. Read Montague.

## Declaration of competing interest

The authors declare no conflict of interest.

## References

[bb0005] Aaltonen K., Näätänen P., Heikkinen M., Koivisto M., Baryshnikov I., Karpov B., Oksanen J., Melartin T., Suominen K., Joffe G., Paunio T., Isometsä E. (2016). Differences and similarities of risk factors for suicidal ideation and attempts among patients with depressive or bipolar disorders. Journal of Affective Disorders.

[bb0010] Ainsworth M.D.S., Blehar M.C., Waters E., Wall S.N. (1978).

[bb0020] Allen J.G. (2011). uilding a therapeutic alliance with the suicidal patient.

[bb0025] Allen J.G. (2013).

[bb0035] Alonso-Arbioll I., Balluerka N., Shaver P.R. (2007). A spanish version of the experiences in close relationships (ECR) adult attachment questionnaire. Personal Relationships.

[bb0040] Anderson J.C., Gerbing D.W. (1988). Structural equation modelling in practice: A review and recommended two-step approach. Psychological Bulletin.

[bb0045] Andrewes H.E., Hulbert C., Cotton S.M., Betts J., Chanen A.M. (2017). An ecological momentary assessment investigation of complex and conflicting emotions in youth with borderline personality disorder. Psychiatry Research.

[bb0050] Angelakis I., Gillespie E.L., Panagioti M. (2019). Childhood maltreatment and adult suicidality: A comprehensive systematic review with meta-analysis. Psychological Medicine.

[bb0055] Angst J., Hengartner M.P., Rogers J., Schnyder U., Steinhausen H.C., Ajdacic-Gross V., Rössler W. (2014). Suicidality in the prospective Zurich study: Prevalence, risk factors and gender. European Archives of Psychiatry and Clinical Neuroscience.

[bb0060] Asarnow J.R., Porta G., Spirito A., Emslie G., Clarke G., Wagner K.D., Vitiello B., Keller M., Birmaher B., McCracken J., Mayes T., Berk M., Brent D.A. (2011). Suicide attempts and nonsuicidal self-injury in the treatment of resistant depression in adolescents: Findings from the TORDIA study. Journal of the American Academy of Child and Adolescent Psychiatry.

[bb0065] Badoud D., Luyten P., Fonseca-Pedrero E., Eliez S., Fonagy P., Debbané M. (2015). The french version of the reflective functioning questionnaire: Validity data for adolescents and adults and its association with non-suicidal self-injury. PLoS ONE.

[bb0070] Barbosa L.P., Quevedo L., da Silva G.D.G., Jansen K., Pinheiro R.T., Branco J.Ô., Lara D., Oses J., da Silva R.A. (2014). Childhood trauma and suicide risk in a sample of young individuals aged 14–35 years in southern Brazil. Child Abuse and Neglect.

[bb0075] Bateman A., Fonagy P. (2008). 8-year follow-up of patients treated for borderline personality disorder: Mentalization-based treatment versus treatment as usual. American Journal of Psychiatry.

[bb0080] Bateman A., Fonagy P. (2009). Randomized controlled trial of outpatient mentalization-based treatment versus structured clinical management for borderline personality disorder. American Journal of Psychiatry.

[bb0085] Bateman A., Fonagy P. (2016). Mentalization-based Treatment for Personality Disorders.

[bb0090] Bateman A.W., Fonagy P. (2004). Mentalization-based treatment of BPD. Journal of Personality Disorders.

[bb0095] Bateman A.W., Fonagy P.E. (2012).

[bb0100] Bernstein D., Fink L. (1998).

[bb0105] Bernstein D.P., Fink L., Handelsman L., Foote J., Lovejoy M., Wenzel K., Sapareto E., Ruggiero J. (1994). Initial reliability and validity of a new retrospective measure of child abuse and neglect. American Journal of Psychiatry.

[bb0110] Berthelot N., Paccalet T., Gilbert E., Moreau I., Mérette C., Gingras N., Rouleau N., Maziade M. (2015). Childhood abuse and neglect may induce deficits in cognitive precursors of psychosis in high-risk children. Journal of Psychiatry & Neuroscience.

[bb0115] Bifulco A., Brown G.W., Lillie A., Jarvis J. (1997). Memories of childhood neglect and abuse: Corroboration in a series of sisters. Journal of Child Psychology and Psychiatry and Allied Disciplines.

[bb0125] Bleiberg E., Rossouw T., Fonagy P. (2012).

[bb0130] Borelli J.L., Ensink K., Hong K., Sereno A.T., Drury R., Fonagy P. (2018). Reflective functioning predicts lower cardiovascular reactivity in school-aged children. Frontiers in Medicine.

[bb0135] Bowlby J. (1969). Loss.

[bb0140] Bretherton I., Munholland K.A., Cassidy J., Shaver P.R. (2008). Handbook of attachment: Theory, research, and clinical applications.

[bb0145] Brewin C.R., Andrews B., Gotlib I.H. (1993). Psychopathology and early experience: A reappraisal of retrospective reports. Psychological Bulletin.

[bb0150] Brown T.A. (2006).

[bb0160] Butchart A., Kahane T., Phinney H.A., Mian M., Furniss T. (2006). Preventing child maltreatment: a guide to taking action and generating evidence.

[bb0165] Byrne B.M. (2005). Factor analytic models: Viewing the structure of an assessment instrument from three perspectives. Journal of Personality Assessment.

[bb0170] Byrne B.M. (2010).

[bb0175] Cassels M., van Harmelen A.-L., Neufeld S., Goodyer I., Jones P.B., Wilkinson P. (2018). Poor family functioning mediates the link between childhood adversity and adolescent nonsuicidal self-injury. Journal of Child Psychology and Psychiatry.

[bb0180] Cecil C.A.M., Zhang Y., Nolte T. (2020). Childhood maltreatment and DNA methylation: A systematic review. Neuroscience and Biobehavioral Reviews.

[bb0190] Chen F.F., Hayes A., Carver C.S., Laurenceau J.P., Zhang Z. (2012). Modeling general and specific variance in multifaceted constructs: A comparison of the bifactor model to other approaches. Journal of Personality.

[bb0195] Chen L., Ngoubene-Atioky A.J., Zanardelli G., Yuanping D., Yu L. (2019). Childhood abuse and suicidal behaviors among Chinese migrant workers: The mediating role of alexithymia and social support. Archives of Suicide Research.

[bb0200] Chiesa M., Fonagy P. (2014). Reflective function as a mediator between childhood adversity, personality disorder and symptom distress. Personality and Mental Health.

[bb0205] Chiesa M., Luyten P., Fonagy P. (2020). Two-year follow-up and changes in reflective functioning in specialist and nonspecialist treatment models for personality disorder. Personality Disorder.

[bb0210] Chou C., Bentler P.M., Satorra A. (1991). Scaled test statistics and robust standard errors for non-normal data in covariance structure analysis: A Monte Carlo study. British Journal of Mathematical and Statistical Psychology.

[bb0215] Coccaro E.F., Berman M.E., Kavoussi R.J. (1997). Assessment of life history of aggression: Development and psychometric characteristics. Psychiatry Research.

[bb0220] Csibra G., Gergely G. (2011). Natural pedagogy as evolutionary adaptation. Philosophical transactions of the Royal Society of LondonSeries B, Biological Sciences.

[bb0225] Curran P.J., West S.G., Finch J.F. (1996). The robustness of test statistics to nonnormality and specification error in confirmatory factor analysis. Psychological Methods.

[bb0230] Derogatis L.R. (1992). The brief symptom inventory (BSI): administration, scoring & procedures manual-II. Clinical PsychometricResearch.

[bb0235] Devi F., Shahwan S., Teh W.L., Sambasivam R., Zhang Y.J., Lau Y.W., Ong S.H., Fung D., Gupta B., Chong S.A., Subramaniam M. (2019). The prevalence of childhood trauma in psychiatric outpatients. Annals of General Psychiatry.

[bb0240] Diamond G., Russon J., Levy S. (2016). Attachment-based family therapy: A review of the empirical support. Family Process.

[bb0245] Diamond G.S., Diamond G.M., Levy S.A. (2014). Attachment-based family therapy for depressed adolescents. American Psychological Association.

[bb0250] Dube S.R., Anda R.F., Felitti V.J., Chapman D.P., Williamson D.F., Giles W.H. (2001). Childhood abuse, household dysfunction, and the risk of attempted suicide throughout the life span: findings from the Adverse Childhood Experiences Study. JAMA.

[bb0255] Dunn K.J., McCray G. (2020). The place of the bifactor model in confirmatory factor analysis investigations into construct dimensionality in language testing. Frontiers in Psychology.

[bb0260] Duñó R., Pousa E., Miguélez M., Montalvo I., Suarez D., Tobeña A. (2009). Suicidality connected with mentalizing anomalies in schizophrenia: A study with stabilized outpatients. Annals of the New York Academy of Sciences.

[bb0265] Duval J., Ensink K., Normandin L., Fonagy P. (2018). Mentalizing mediates the association between childhood maltreatment and adolescent borderline and narcissistic personality traits. Adolescent Psychiatry.

[bb0270] Ensink K., Bégin M., Normandin L., Godbout N., Fonagy P. (2017). Mentalization and dissociation in the context of trauma: Implications for child psychopathology. Journal of Trauma and Dissociation.

[bb0275] Espeleta H.C., Palasciano-Barton S., Messman-Moore T.L. (2017). The impact of child abuse severity on adult attachment anxiety and avoidance in college women: The role of emotion dysregulation. Journal of Family Violence.

[bb0280] Euler S., Nolte T., Constantinou M., Griem J., Montague P.R., Fonagy P., Personality and Mood Disorders Research Network (2019). Interpersonal problems in borderline personality disorder: associations with mentalizing, emotion regulation, and impulsiveness. Journal of Personality Disorders.

[bb0285] Felitti V.J., Anda R.F., Nordenberg D., Williamson D.F., Spitz A.M., Edwards V., Marks J.S. (1998). Relationship of childhood abuse and household dysfunction to many of the leading causes of death in adults: The Adverse Childhood Experiences (ACE) Study. American Journal of Preventive Medicine.

[bb0290] First M.B. (2014). The encyclopedia of clinical psychology.

[bb0295] Fonagy P., Allison E. (2014). The role of mentalizing and epistemic trust in the therapeutic relationship. Psychotherapy.

[bb0300] Fonagy P., Bateman A.W. (2006). Mechanisms of change in mentalization-based treatment of BPD. Journal of Clinical Psychology.

[bb0310] Fonagy P., Bateman A.W. (2016). Adversity, attachment, and mentalizing. Comprehensive Psychiatry.

[bb0320] Fonagy P., Luyten P. (2016).

[bb0325] Fonagy P., Target M. (1997). Attachment and reflective function: Their role in self-organization. Development and Psychopathology.

[bb0340] Fonagy P., Target M., Steele H., Steele M. (1998). Reflective-functioning manual, version 5.0, for application to adult attachment interviews.

[bb0345] Fonagy P., Gergely G., Jurist E.L., Target M. (2002). Affect regulation, mentalization and the development of the self.

[bb0350] Fonagy P., Luyten P., Allison E. (2015). Epistemic petrification and the restoration of epistemic trust: A new conceptualization of borderline personality disorder and its psychosocial treatment. Journal of Personality Disorders.

[bb0355] Fonagy P., Luyten P., Moulton-Perkins A., Lee Y.W., Warren F., Howard S., Ghinai R., Fearon P., Lowyck B. (2016). Development and validation of a self-report measure of mentalizing: The reflective functioning questionnaire. PLoS ONE.

[bb0360] Fonagy P., Campbell C., Bateman A. (2017). Mentalizing, attachment, and epistemic trust in group therapy. International Journal of Group Psychotherapy.

[bb0365] Fonagy P., Luyten P., Allison E., Campbell C. (2019). Mentalizing, epistemic trust and the phenomenology of psychotherapy. Psychopathology.

[bb0370] Forrester R.L., Slater H., Jomar K., Mitzman S., Taylor P.J. (2017). Self-esteem and non-suicidal self-injury in adulthood: A systematic review. Journal of Affective Disorders.

[bb0375] Fraley R.C., Shaver P.R. (2000). Adult romantic attachment: Theoretical developments, emerging controversies, and unanswered questions. Review of General Psychology.

[bb0380] Fraley R.C., Waller N.G., Brennan K.A. (2000). An item response theory analysis of self-report measures of adult attachment. Journal of Personality and Social Psychology.

[bb0385] Franzke I., Wabnitz P., Catani C. (2015). Dissociation as a mediator of the relationship between childhood trauma and nonsuicidal self-injury in females: A path analytic approach. Journal of Trauma and Dissociation.

[bb0390] George C., Kaplan N., Main M. (1996).

[bb0395] Germine L., Dunn E.C., McLaughlin K.A., Smoller J.W. (2015). Childhood adversity is associated with adult theory of mind and social affiliation, but not face processing. PLoS One.

[bb0400] Glassman L.H., Weierich M.R., Hooley J.M., Deliberto T.L., Nock M.K. (2007). Child maltreatment, non-suicidal self-injury, and the mediating role of self-criticism. Behaviour Research and Therapy.

[bb0405] Grady M.D., Looman J., Abracen J. (2019). Childhood abuse, attachment, and psychopathy among individuals who commit sexual offenses. Sexual Addiction and Compulsivity.

[bb0410] Granqvist P., Sroufe L.A., Dozier M., Hesse E., Steele M., van Ijzendoorn M., Solomon J., Schuengel C., Fearon P., Bakermans-Kranenburg M., Steele H., Cassidy J., Carlson E., Madigan S., Jacobvitz D., Foster S., Behrens K., Rifkin-Graboi A., Gribneau N., Duschinsky R. (2017). Disorganized attachment in infancy: a review of the phenomenon and its implications for clinicians and policy-makers. Attachment & Human Development.

[bb0415] Green B.L., Grace M.C., Vary M.G., Kramer T.L., Gleser G.C., Leonard A.C. (1994). Children of disaster in the second decade: A 17-year follow-up of Buffalo Creek survivors. Journal of the American Academy of Child & Adolescent Psychiatry.

[bb0420] Grunebaum M.F., Galfalvy H.C., Mortenson L.Y., Burke A.K., Oquendo M.A., Mann J.J. (2010). Attachment and social adjustment: Relationships to suicide attempt and major depressive episode in a prospective study. Journal of Affective Disorders.

[bb0425] Hankin B.L. (2005). Childhood maltreatment and psychopathology: Prospective tests of attachment, cognitive vulnerability, and stress as mediating processes. Cognitive Therapy and Research.

[bb0430] Hanson J.L., van den Bos W., Roeber B.J., Rudolph K.D., Davidson R.J., Pollak S.D. (2017). Early adversity and learning: implications for typical and atypical behavioral development. Journal of Child Psychology and Psychiatry.

[bb0435] Hardt J., Rutter M. (2004). Validity of adult retrospective reports of adverse childhood experiences: Review of the evidence. Journal of Child Psychology and Psychiatry and Allied Disciplines.

[bb0440] Hatkevich C., Venta A., Sharp C. (2019). Theory of mind and suicide ideation and attempt in adolescent inpatients. Journal of Affective Disorders.

[bb0445] Hazan C., Shaver P. (1987). Romantic love conceptualized as an attachment process. Journal of Personality and Social Psychology.

[bb0450] Hollerbach P., Johansson A., Ventus D., Jern P., Neumann C.S., Westberg L., Santtila P., Habermeyer E., Mokros A. (2018). Main and interaction effects of childhood trauma and the MAOA uVNTR polymorphism on psychopathy. Psychoneuroendocrinology.

[bb0455] Hu L.T., Bentler P.M. (1999). Cutoff criteria for fit indexes in covariance structure analysis: Conventional criteria versus new alternatives. Structural Equation Modeling.

[bb0460] Huang Y.L., Fonagy P., Feigenbaum J., Montague P.R., Nolte T. (2020). Multidirectional Pathways between Attachment, Mentalizing, and Posttraumatic Stress Symptomatology in the Context of Childhood Trauma. Psychopathology.

[bb0465] Kaess M. (2019). Differential pathways from childhood maltreatment to self-harm and suicidal ideation. European Child and Adolescent Psychiatry.

[bb0470] Kemberg O.F., Yeomans F.E., Clarkin J.F., Levy K.N. (2008). Transference focused psychotherapy: Overview and update. International Journal of Psychoanalysis.

[bb0475] Kline R.B. (2016).

[bb0480] Klonsky E.D., May A.M., Saffer B.Y. (2016). Suicide, suicide attempts, and suicidal ideation. Annual Review of Clinical Psychology.

[bb0485] Kongerslev M.T., Bach B., Rossi G., Trauelsen A.M., Ladegaard N., Løkkegaard S.S., Bo S. (2019). Psychometric validation of the Childhood Trauma Questionnaire-Short Form (CTQ-SF) in a Danish clinical sample. Child Abuse and Neglect.

[bb0490] Kristiansen V.R., Handeland T.B., Lau B., Søderstrøm K., Håkansson U., Øie M.G. (2020). Trauma in childhood and adolescence and impaired executive functions are associated with uncertain reflective functioning in mothers with substance use disorder. Addictive Behaviors Reports.

[bb0495] Labonte B., Turecki G. (2010). The epigenetics of suicide: Explaining the biological effects of early life environmental adversity. Archives of Suicide Research.

[bb0505] Lang C.M., Sharma-Patel K. (2011). The relation between childhood maltreatment and self-injury: A review of the literature on conceptualization and intervention. Trauma, Violence, and Abuse.

[bb0515] Lassri D., Luyten P., Fonagy P., Shahar G. (2018). Undetected scars? Self-criticism, attachment, and romantic relationships among otherwise well-functioning childhood sexual abuse survivors. Psychological Trauma: Theory, Research, Practice, and Policy.

[bb0520] Lee M.A. (2015). Emotional abuse in childhood and suicidality: The mediating roles of re-victimization and depressive symptoms in adulthood. Child Abuse and Neglect.

[bb0525] Lee S., Fung S.C., Tsang A., Liu Z.R., Huang Y.Q., He Y.L., Zhang M.Y., Shen Y.C., Nock M.K., Kessler R.C. (2007). Lifetime prevalence of suicide ideation, plan, and attempt in metropolitan China. Acta Psychiatrica Scandinavica.

[bb0530] Lemaigre C., Taylor E.P. (2019). Mediators of childhood trauma and suicidality in a cohort of socio-economically deprived Scottish men. Child Abuse and Neglect.

[bb0535] Levesque C., Lafontaine M.F., Bureau J.F., Cloutier P., Dandurand C. (2010). The influence of romantic attachment and intimate partner violence on non-suicidal self-injury in young adults. Journal of Youth and Adolescence.

[bb0540] Levi-Belz Y., Gvion Y., Horesh N., Apter A. (2013). Attachment patterns in medically serious suicide attempts: The mediating role of self-disclosure and loneliness. Suicide and Life-threatening Behavior.

[bb0545] Levy K.N., Yeomans F.E., Diamond D. (2007). Psychodynamic treatments of self-injury. Journal of Clinical Psychology.

[bb0550] Li C.H. (2016). Confirmatory factor analysis with ordinal data: Comparing robust maximum likelihood and diagonally weighted least squares. Behavior Research Methods.

[bb0555] Li C.-H. (2014). The performance of MLR, USLMV, and WLSMV estimation in structural regression models with ordinal variables. Journal of Chemical Information and Modeling.

[bb0560] Liu J., Fang Y., Gong J., Cui X., Meng T., Xiao B., He Y., Shen Y., Luo X. (2017). Associations between suicidal behavior and childhood abuse and neglect: A meta-analysis. Journal of Affective Disorders.

[bb0565] Liu R.T., Scopelliti K.M., Pittman S.K., Zamora A.S. (2018). Childhood maltreatment and non-suicidal self-injury: A systematic review and meta-analysis. The Lancet Psychiatry.

[bb0570] Lopez-Castro T., Saraiya T., Zumberg-Smith K., Dambreville N. (2019). Association between shame and posttraumatic stress disorder: A meta-analysis. Journal of Traumatic Stress.

[bb0575] Luyten P., Fonagy P., Lowyck B., Vermote R., Fonagy P., Luyten, Bateman A.W., Fonagy P.E. (2012). Handbook of mentalizing in mental health practice.

[bb0580] Luyten P., Campbell C., Fonagy P. (2020). Borderline personality disorder, complex trauma, and problems with self and identity: A social-communicative approach. Journal of Personality.

[bb0585] Martin J., Raby K.L., Labella M.H., Roisman G.I. (2017). Childhood abuse and neglect, attachment states of mind, and non-suicidal self-injury. Attachment and Human Development.

[bb0590] McConnell M., Moss E. (2011). Attachment across the life span: Factors that contribute to stability and change. Australian Journal of Educational & Developmental Psychology.

[bb0595] McCrory E., Viding E. (2015). The theory of latent vulnerability: Reconceptualizing the link between childhood maltreatment and psychiatric disorder. Development and Psychopathology.

[bb0600] Mickelson K.D., Kessler R.C., Shaver P.R. (1997). Adult attachment in a nationally representative sample. Journal of Personality and Social Psychology.

[bb0610] Mikulincer M., Shaver P.R. (2016). Structure, dynamics and change.

[bb0620] Morey L.C. (1991).

[bb0625] Mou D., Kleiman E.M., Fedor S., Beck S., Huffman J.C., Nock M.K. (2018). Negative affect is more strongly associated with suicidal thinking among suicidal patients with borderline personality disorder than those without. Journal of Psychiatric Research.

[bb0630] Murphy A., Steele M., Dube S.R., Bate J., Bonuck K., Meissner P., Goldman H., Steele H. (2014). Adverse childhood experiences (ACEs) questionnaire and adult attachment interview (AAI): Implications for parent child relationships. Child Abuse & Neglect.

[bb0635] Nock M.K., Favazza A.R. (2009). Understanding nonsuicidal self-injury: Origins, assessment, and treatment.

[bb0640] Nock M.K., Joiner T.E.B., Gordon K.H., Lloyd-Richardson E., Prinstein M.J. (2006). Non-suicidal self-injury among adolescents: Diagnostic correlates and relation to suicide attempts. Psychiatry Research.

[bb0645] Nolte T., Bolling D.Z., Hudac C.M., Fonagy P., Mayes L., Pelphrey K.A. (2013). Brain mechanisms underlying the impact of attachment-related stress on social cognition. Frontiers in Human Neuroscience.

[bb0650] Paul E., Ortin A. (2019). Psychopathological mechanisms of early neglect and abuse on suicidal ideation and self-harm in middle childhood. European Child and Adolescent Psychiatry.

[bb0655] Podsakoff P.M., MacKenzie S.B., Lee J.Y., Podsakoff N.P. (2003). Common method biases in behavioral research: a critical review of the literature and recommended remedies. Journal of Applied Psychology.

[bb0660] Quek J., Newman L.K., Bennett C., Gordon M.S., Saeedi N., Melvin G.A. (2017). Reflective function mediates the relationship between emotional maltreatment and borderline pathology in adolescents: A preliminary investigation. Child Abuse and Neglect.

[bb0665] Raby K.L., Labella M.H., Martin J., Carlson E.A., Roisman G.I. (2017). Childhood abuse and neglect and insecure attachment states of mind in adulthood: Prospective, longitudinal evidence from a high-risk sample. Development and Psychopathology.

[bb0670] Reichl C., Kaess M. (2021). Self-harm in the context of borderline personality disorder. Current Opinion in Psychology.

[bb0675] Reise S.P., Moore T.M., Haviland M.G. (2010). Bifactor Models and Rotations. Journal of Personality Assessment.

[bb0680] Rogers M.L., Ringer F.B., Joiner T.E. (2018). The association between suicidal ideation and lifetime suicide attempts is strongest at low levels of depression. Psychiatry Research.

[bb0685] Rossouw T.I., Fonagy P. (2012). Mentalization-based treatment for self-harm in adolescents: A randomized controlled trial. Journal of the American Academy of Child and Adolescent Psychiatry.

[bb0690] Sachs-Ericsson N.J., Rushing N.C., Stanley I.H., Sheffler J. (2016). In my end is my beginning: Developmental trajectories of adverse childhood experiences to late-life suicide. Aging and Mental Health.

[bb0695] Satorra C., Bentler P.M., von Eye A., Clogg C.C. (1994). Latent variable analysis: Applications for developmental research.

[bb0700] Scala J.W., Levy K.N., Johnson B.N., Kivity Y., Ellison W.D., Pincus A.L., Wilson S.J., Newman M.G. (2018). The role of negative affect and self-concept clarity in predicting self-injurious urges in borderline personality disorder using ecological momentary assessment. Journal of Personality Disorders.

[bb0705] Sibley C.G., Fischer R., Liu J.H. (2005). Reliability and Validity of the Revised Experiences in Close Relationships (ECR-R) Self-Report Measure of Adult Romantic Attachment. Personality and Social Psychology Bulletin.

[bb0710] Simon M., Németh N., Gálber M., Lakner E., Csernela E., Tényi T., Czéh B. (2019). Childhood adversity impairs theory of mind abilities in adult patients with major depressive disorder. Frontiers in Psychiatry.

[bb0715] Smith P.N., Gamble S.A., Cort N.A., Ward E.A., Conwell Y., Talbot N.L. (2012). The relationships of attachment style and social maladjustment to death ideation in depressed women with a history of childhood sexual abuse. Journal of Clinical Psychology.

[bb0720] Sperber D., Clément F., Heintz C., Mascaro O., Mercier H., Origgi G., Wilson D. (2010). Epistemic vigilance. Mind & Language.

[bb0725] Spinhoven P., Penninx B.W., Hickendorff M., van Hemert A.M., Bernstein D.P., Elzinga B.M. (2014). Childhood Trauma Questionnaire: factor structure, measurement invariance, and validity across emotional disorders. Psychological Assessment.

[bb0730] Spitzer C., Zimmermann J., Brähler E., Euler S., Wendt L., Müller S. (2021). The German version of the Reflective Functioning Questionnaire (RFQ): A statistical test review in the general population. PPmP Psychotherapy Psychosomatics Medical Psychology.

[bb0735] Tavakol M., Dennick R. (2011). Making sense of Cronbach’s alpha. International journal of Medical Education.

[bb0740] Taylor P.J., Jomar K., Dhingra K., Forrester R., Shahmalak U., Dickson J.M. (2018). A meta-analysis of the prevalence of different functions of non-suicidal self-injury. Journal of Affective Disorders.

[bb0745] Tényi T., Czéh B. (2019). Childhood adversity impairs theory of mind abilities in adult patients with major depressive disorder. Frontiers in Psychiatry.

[bb0750] Thombs B.D., Bernstein D.P., Lobbestael J., Arntz A. (2009). A validation study of the Dutch Childhood Trauma Questionnaire-Short Form: Factor structure, reliability, and known-groups validity. Child Abuse and Neglect.

[bb0755] Titelius E.N., Cook E., Spas J., Orchowski L., Kivisto K., O’Brien K., Frazier E., Wolff J.C., Dickstein D.P., Kim K.L., Seymour K.E. (2018). Emotion Dysregulation Mediates the Relationship Between Child Maltreatment and Non-Suicidal Self-injury. Journal of Aggression, Maltreatment and Trauma.

[bb0760] Waters E., Merrick S., Treboux D., Crowell J., Albersheim L. (2000). Attachment security in infancy and early adulthood: A twenty-year longitudinal study. Child Development.

[bb0765] Weijers J., Fonagy P., Eurelings-Bontekoe E., Termorshuizen F., Viechtbauer W., Selten J.P. (2018). Mentalizing impairment as a mediator between reported childhood abuse and outcome in nonaffective psychotic disorder. Psychiatry Research.

[bb0770] Wendt L.P., Wright A.G.C., Pilkonis P.A., Nolte T., Fonagy P., Montague P.R., Benecke C., Krieger T., Zimmermann J. (2019). The latent structure of interpersonal problems: Validity of dimensional, categorical, and hybrid models. Journal of Abnormal Psychology.

[bb0775] Whitlock J., Eckenrode J., Silverman D. (2006). Self-injurious behaviors in a college population. Pediatrics.

[bb0780] Wilkinson P., Kelvin R., Roberts C., Dubicka B., Goodyer I. (2011). Clinical and psychosocial predictors of suicide attempts and nonsuicidal self-injury in the Adolescent Depression Antidepressants and Psychotherapy Trial (ADAPT). American Journal of Psychiatry.

[bb0785] Wilkinson P.O., Qiu T., Neufeld S., Jones P.B., Goodyer I.M. (2018). Sporadic and recurrent non-suicidal self-injury before age 14 and incident onset of psychiatric disorders by 17 years: Prospective cohort study. British Journal of Psychiatry.

[bb0790] World Health Organization (2014).

[bb0795] World Health Organization (2019).

[bb0800] Wrath A.J., Adams G.C. (2019). Self-Injurious Behaviors and Adult Attachment: A Review of the Literature. Archives of Suicide Research.

[bb0805] Yates T.M., Carlson E.A., Egeland B. (2008). A prospective study of child maltreatment and self-injurious behavior in a community sample. Development and Psychopathology.

[bb0810] Zatti C., Rosa V., Barros A., Valdivia L., Calegaro V.C., Freitas L.H., Ceresér K.M.M., da Rocha N.S., Bastos A.G., Schuch F.B. (2017). Childhood trauma and suicide attempt: A meta-analysis of longitudinal studies from the last decade. Psychiatry Research.

[bb0815] Zayas V., Mischel W., Shoda Y., Aber J.L. (2011). Roots of adult attachment: Maternal caregiving at 18 months predicts adult peer and partner attachment. Social Psychological and Personality Science.

[bb0820] Zietlow A.L., Nonnenmacher N., Reck C., Mueller M., Herpertz S.C., Neukel C., Fuchs A., Bermpohl F., Fuehrer D., Kluczniok D., Attar C.H., Jaite C., Dittrich K., Boedeker K. (2017). Early life maltreatment but not lifetime depression predicts insecure attachment in women. Archives of Women’s Mental Health.

[bb0825] Zortea T.C., Gray C.M., O’Connor R.C. (2019). The relationship between adult attachment and suicidal thoughts and behaviors: A systematic review. Archives of Suicide Research.

